# Supplementary material for: MetaRibo-Seq measures translation in microbiomes
Source: Nat Commun. 2020 Jun 29;11:3268. doi: 10.1038/s41467-020-17081-z (PMC7324362; doi:10.1038/s41467-020-17081-z)
Supplement: Supplementary file 10 — Supplementary Data 7 [file 41467_2020_17081_MOESM10_ESM.zip › File2/Confidence_High_Taxonomy/2363_out.krona.html]

Javascript must be enabled to view this page.

members
magnitude
magnitudeUnassigned
count
unassigned
taxon
rank

2363\_out

6


SRS013476\_contig\_number\_contig-100\_25992.25992SRS024132\_contig\_number\_contig-100\_10100.214676SRS146812\_contig\_number\_contig-100\_37065.37065
3

superkingdom
3
2

phylum
3
1239

186801
1
class

186802
order
1

family
1
216572

genus
1
459786


SRS017916\_contig\_number\_13185
1945593
1
species

species
2

SRS077231\_contig\_number\_20275SRS078419\_contig\_number\_8938
1897031
